# Posttraumatic stress disorder and prolonged grief in refugees exposed to trauma and loss

**DOI:** 10.1186/1471-244X-14-106

**Published:** 2014-04-09

**Authors:** Angela Nickerson, Belinda J Liddell, Fiona Maccallum, Zachary Steel, Derrick Silove, Richard A Bryant

**Affiliations:** 1 School of Psychology, University of New South Wales, Sydney NSW 2052, Australia; 2 Psychiatry Research and Teaching Unit, School of Psychiatry, University of New South Wales, Sydney NSW, 2052, Australia

**Keywords:** Trauma, Bereavement, Posttraumatic stress disorder, Grief, Refugees, War

## Abstract

**Background:**

While a large proportion of conflict-affected populations have been dually exposed to trauma and loss, there is inadequate research identifying differential symptom profiles related to bereavement and trauma exposure in these groups. The objective of this study were to (1) determine whether there are distinct classes of posttraumatic stress disorder (PTSD) and prolonged grief disorder (PGD) symptoms in bereaved trauma survivors exposed to conflict and persecution, and (2) examine whether particular types of refugee experiences and stressors differentially predict symptom profiles.

**Methods:**

Participants were 248 Mandaean adult refugees who were assessed at an average of 4.3 years since entering Australia following persecution in Iraq. PTSD, PGD, trauma exposure, adjustment difficulties since relocation, and English proficiency were measured. Latent class analysis was used to elucidate symptom profiles of PTSD and PGD in this sample.

**Results:**

Latent class analysis revealed four classes of participants: a combined PTSD/PGD class (16%), a predominantly PTSD class (25%), a predominantly PGD class (16%), and a resilient class (43%). Whereas membership in the PTSD/PGD class was predicted by exposure to traumatic loss, those in the PGD class were more likely to have experienced adaptation difficulties since relocation, and individuals in the PTSD class were more likely to have experienced difficulties related to loss of culture and support.

**Conclusions:**

This study provides evidence that specific symptom patterns emerge following exposure to mass trauma and loss. These profiles are associated with distinct types of traumatic experiences and post-migration living difficulties. These results have substantial public health implications for assessment and intervention following mass trauma.

## Background

A large proportion of the world’s population is exposed to trauma and loss in the context of conflict and persecution, as evidenced by the fact that there have been over 200 armed conflicts in 151 locations since World War II [1]. The high prevalence of posttraumatic stress disorder (PTSD) amongst conflict-affected and displaced populations has been well-documented [2,3]. While trauma in these contexts also often involves the death of family and friends, relatively less research has investigated the impact of bereavement in conflict-affected and displaced populations. Grief is an expected reaction following the loss of a loved one; and research suggests that grief responses that persist beyond six to twelve months after the loss may be indicative of prolonged grief disorder (PGD) [4]. The diagnosis of PGD is being considered for inclusion in *ICD-11*[5]. This condition describes chronic maladaptive grief responses, with a persistent sense of longing or yearning for the deceased being central to the diagnosis [6]. PGD has been reported in approximately 10% of bereaved individuals [6,7], although higher rates have also been observed amongst refugee and other non-western conflict-affected populations [8-10].

To date, there has been no investigation of how PTSD and PGD symptom profiles manifest in populations exposed to violence–related loss and trauma. An outstanding question is the extent to which groups exposed to multiple traumas and losses show different PTSD and/or PGD symptom patterns compared to bereaved groups exposed to largely single-incident civilian trauma. Studies investigating western traumatized populations have identified three sub-groups of trauma survivors characterized by levels of PTSD symptoms; namely pervasive disturbance, moderate disturbance or no disturbance [11-13]. It may be that these findings (derived from research with trauma-exposed individuals) generalize to conflict and persecution-exposed groups (who are typically exposed to both trauma and loss), such that symptom profiles in these groups are characterized by high, moderate or no disturbance across both PTSD and PGD symptoms. If this is the case, it would indicate that the dual impact of loss and trauma exerts a pervasively negative effect on mental health, without differentiating between PTSD and PGD symptoms. Alternatively, distinct PTSD or PGD symptom profiles may emerge, indicating that these responses are distinguishable even in a highly traumatized and bereaved population. Identifying specific factors that are associated with distinct symptom clusters would also yield important information regarding profiles of distress following exposure to both trauma and loss.

We employed latent class analysis (LCA) to investigate whether subpopulations characterized by differential symptom profiles of PTSD and PGD could be identified in a refugee sample exposed to both significant trauma and loss, and whether specific refugee experiences predicted different symptom profiles. The sample for this study was drawn from a community of Iraqi Mandaean refugees residing in Sydney, Australia. The Mandaeans are a gnostic sect originating in Iraq and Iran who have been subjected to repeated traumatization and suffered many losses [14,15].

## Method

### Participants

Participants were 248 adults from the Mandaean community residing in Sydney, Australia who reported that they had lost a loved one. While no census information was available, community leaders identified a potential testing sample of 367 individuals based on existing community lists. Of these, 52 individuals could not be contacted or refused to participate, and 67 had not been bereaved (86% response rate).

### Measures

The Harvard Trauma Questionnaire [16] was used to assess trauma exposure and PTSD symptoms. The trauma exposure sub-scale encompasses 16 types of traumatic events, while the PTSD sub-scale indexes 16 PTSD symptoms. We used categories of traumatic experiences that were empirically derived by Steel and colleagues [17] with a sample of refugees, asylum-seekers and migrants. Each subscale represented a count of the number of types of trauma in each domain experienced by the individual. Subscales encompassed detention and abuse (range = 0 to 8), traumatic loss (range = 0 to 3) and exposure to conflict (range = 0 to 5). We also derived dichotomous indicator variables for each PTSD symptom (symptom absent/symptom present). A symptom was considered to be present if the individual rated it as bothering them “quite a bit” (3) or “extremely” (4).

The Inventory of Complicated Grief [18] measured symptoms of PGD. This 12-item scale encompasses nine items indexing symptoms proposed for the diagnosis of PGD; and three items relating to duration of symptoms and impairment. Only the nine symptom items were used in this study to quantify the presence and severity of PGD symptoms. A dichotomous indicator variable for each symptom was derived for the present study (symptom absent/present). A symptom was considered to be present if the individual rated it as occurring “*sometimes*” (3), “*often*” (4), or “*always*” (5) or experiencing it as “*some*” (3), “*marked*” (4) or “*overwhelming*” (5).

The Hopkins Symptom Checklist-Depression Subscale [19] was used to measure symptoms of depression. This 15-item subscale provides a continuous measure of symptoms of depression (with a range from 0 to 60) and diagnostic caseness by either applying cut-off score or a DSM-IV-derived algorithm (Mollica et al., 2001). In this study, the DSM-IV derived algorithm was used to represent depression caseness.

The Post Migration Living Difficulties Checklist [17,20] was used to assess daily living difficulties. This 19-item scale examines the extent to which post-migration challenges had been of concern over the past twelve months. Items scored as “*a serious problem”* or “*a very serious problem”* were considered positive responses. We used categories of post-migration living difficulty experiences that were empirically derived by Steel and colleagues [17] with a sample of refugees, asylum-seekers and migrants. These encompassed adaptation difficulties (range = 0 to 7), threat to family (range = 0 to 2), residency determination difficulties (range = 0 to 3), health, welfare and asylum difficulties (range = 0 to 4) and loss of culture and support (range = 0 to 7). The score on each subscale was represented by a count of items scored as positive in the pertinent domain.

The International Second Language Proficiency Rating Scale [21] was used to measure English language competency. This scale yields a continuous score ranging from 0 (no ability to speak English) to 7 (communicating as a native speaker).

### Procedure

No census data was available for the Mandaeans in Sydney when this study was conducted however information was derived from multiple sources (community leaders, community members, and service providers) to suggest that there were approximately 600 Arabic-speaking adult Mandaeans residing in Sydney at the time of this study. Community leaders provided lists of potential participants, which numbered 367 adults. One of three bilingual (Arabic and English-speaking) Mandaean research assistants invited potential participants to take part in this study by telephone. Fifty-two persons declined to participate, resulting in a sample of 315 Mandaeans. 248 individuals reported that they had lost a loved one, and were included in the current study. This represents a response rate of 86% and an estimated coverage of adult Mandaeans in Sydney of 53%.

The three research assistants administered the interviews in this study in the participants’ homes. These research assistants received two days of training on the administration of mental health measures, and received weekly supervision from the first author. Participants were provided with $AUD50 payment. All measures were translated into Arabic and back-translated into English (by translators blind to the original version) using gold-standard procedures, and discrepancies resolved by the research team and translators [22]. After the nature of the study was explained, written informed consent was obtained for all participants. All measures were administered in interview form, with the interviews lasting between 45 minutes and 75 minutes. This study had ethical approval from the University of New South Wales Research Ethics Committee. Data was collected between September 2006 and November 2007.

### Statistical analysis

LCA was used to model PTSD and PGD symptom profiles, using Mplus v.6 [23]. LCA uses binary indicators to identify patterns of responses, assigning individuals to classes on the basis of these patterns. We identified latent classes on the basis of dichotomous indicators of PTSD and PGD symptoms. Full maximum likelihood estimation was used to adjust for missing data on latent class indicator variables. LCA identifies the minimum number of classes that can account for associations between symptoms. The most parsimonious (one-class) model was initially fitted, followed by successive models with increasing numbers of classes to determine the number of latent classes that best fit the data. We assessed comparative model fit using the following indices: Sample-Size Adjusted Bayesian Information Criterion (SS-BIC), the Akaike’s Information Criterion (AIC), and entropy. Better fit is evidenced by lower values of the SS-BIC and AIC, and higher values of entropy. We also considered parsimony and interpretability when evaluating the optimal class solution.

To assess predictors of class membership, we conducted multinomial logistic regression analyses in SPSS version 20. Class membership was derived from the optimal latent class model. Predictors included in the initial model were age; gender; English proficiency; exposure to detention and abuse, traumatic loss, and conflict; and adaptation difficulties, threat to family, residency determination difficulties, healthcare, welfare, asylum difficulties, and loss of culture and support. We also assessed the association between class membership and symptoms of depression (using the continuous measure of depression symptoms derived from the HSCL), and calculated the percentage of individuals in each class that met DSM-IV criteria for depression (using the DSM-IV-derived algorithm to determine caseness from the HSCL).

## Results

### Participants & exposure to trauma and living difficulties

Participants were 48% male, with a mean age of 38.31 years (*SD* = 14.53), and length of education of 10.98 years (*SD* = 3.78). Participants had been in Australia for an average of 4.31 years (*SD* = 4.25). Information on sample exposure to traumatic events and living difficulties is presented in Tables 1 and 2, respectively.

**Table 1:** Exposure to traumatic events

	**Mean/N**	**SD/%**
Overall trauma exposure (mean number of types)	4.01	3.32
Detention and abuse exposure (mean number of types)	1.65	1.70
Ill health without access to medical care	78	31.45%
Imprisonment	53	21.37%
Rape or sexual abuse	7	2.82%
Forced isolation from others	29	11.69%
Being close to death	146	58.87%
Forced separation from family members	40	16.13%
Lost or kidnapped	29	11.69%
Torture	24	9.68%
Traumatic loss exposure (mean number of types)	1.24	1.15
Murder of family or friends	114	45.97%
Unnatural death of family or friends	120	48.39%
Murder of stranger or strangers	70	28.23%
Exposure to conflict (mean number of types)	1.16	1.23
Lack of food or water	120	48.39%
Lack of shelter	76	30.65%
Serious injury	30	12.10%
Combat situation	48	19.35%
Brainwashing	11	4.43%

**Table 2:** Exposure to living difficulties

	**Mean/N**	**SD/%**
Overall living difficulties exposure (mean number of types)	7.91	4.81
Residency determination difficulties (mean number of types)	0.36	0.54
Interviews with immigration officials	7	2.82%
Conflict with immigration officials	4	1.61%
Fears of being sent home	80	32.26%
Healthcare, welfare and asylum difficulties (mean number of types)	0.98	1.06
Poor access to medical care	77	31.04%
Poor access to long-term medical care	77	31.04%
Poor access to dentistry care	97	39.11%
Poor access to counselling services	75	30.24%
Little government help with welfare	31	12.50%
Little help with welfare from charities	27	10.89%
Threat to Family (mean number of types)	1.85	0.97
Separation from family	76	30.65%
Worries about family back at home	201	81.04%
Unable to return home in emergency	163	65.73%
Adaptation difficulties (mean number of types)	1.60	1.27
Communication difficulties	130	52.42%
Discrimination	12	4.84%
Not being able to find work	97	39.11%
Bad job conditions	35	14.11%
Poverty	109	43.96%
Loss of culture and support (mean number of types)	1.30	1.46
Loneliness and boredom	113	45.56%
Isolation	95	38.31%
Lack of access to foods appropriate for your religion	75	30.24%
Lack of access to places where you can conduct religious ceremonies	128	51.61%
Refused permission to perform necessary religious rituals	63	25.40%
Lack of organized social activities for your community	56	22.5%
People not recognizing your religion as a legitimate religion	44	17.74%

### Latent class analysis

**Figure 1:**
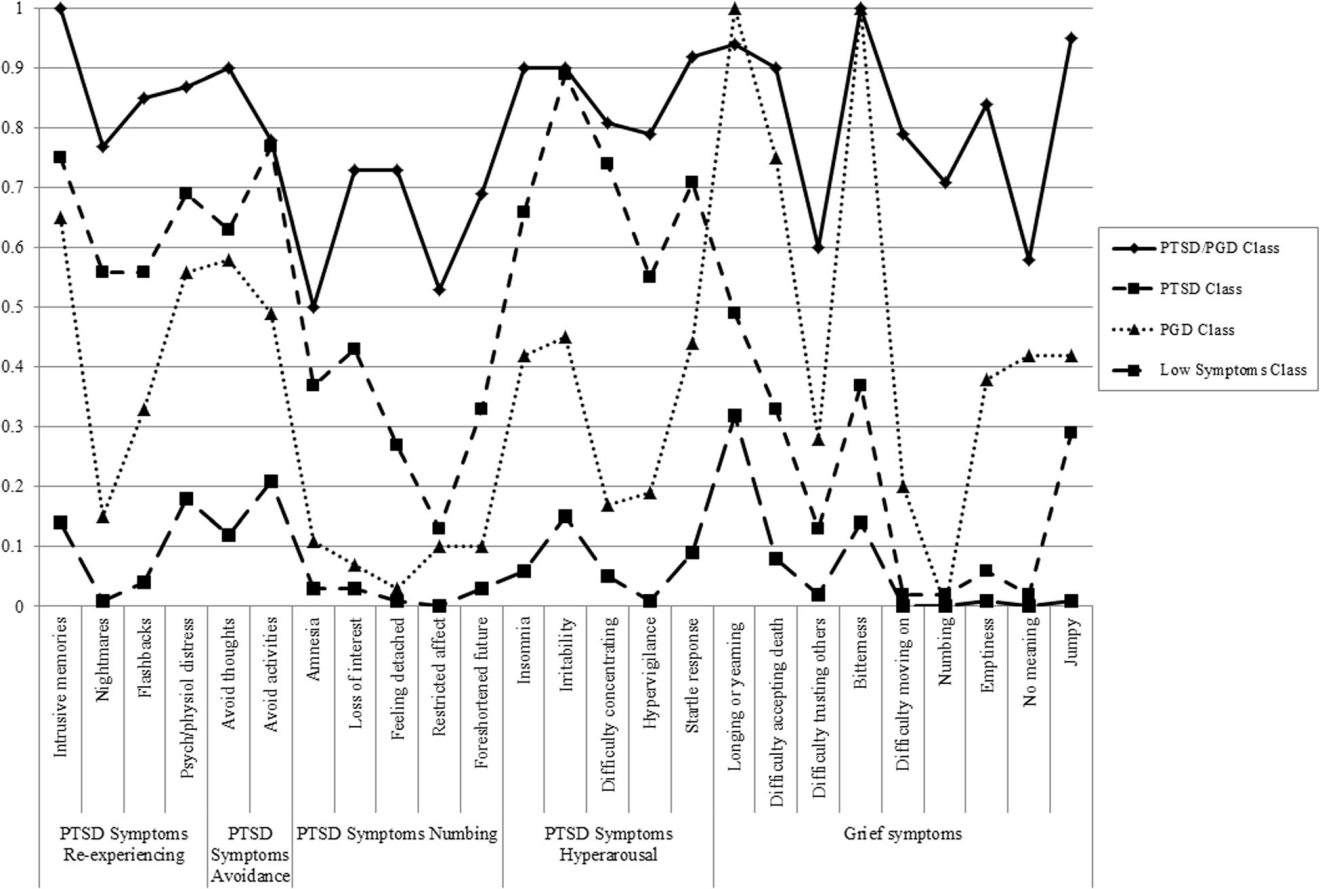
Estimated symptom prevalence for four-class solution.

The goodness-of-fit indices for the one to five class models are presented in Table 3. Based on fit indices and interpretability of class solutions, a four-class solution was judged to be the optimal solution. This solution comprises a combined PTSD/PGD class (16%), a predominantly PTSD class (25%), a predominantly PGD class (16%), and a Resilient class (43%). While a five-class solution yielded marginally lower Loglikelihood, SSBIC and AIC indices than the four-class solution, the entropy value was lower. Further, the additional fifth class was not clearly distinguishable from the other classes. Thus the more parsimonious four-class solution was retained. Overall symptom prevalence rates and conditional probabilities of symptoms for the four-class solution are reported in Table 4. The estimated symptom probabilities for each of the four classes are presented in Figure 1, representing the percentage membership in each class exhibiting each PTSD and PGD symptom. We considered values of ≥ .60 as representing high probability, values ≤ .59 and ≥ .15 as representing a moderate probability and values of ≤ .15 representing a low probability that the symptom was present in the class [24].

**Table 3:** Goodness-of-fit statistics for 1 to 6 class solutions

**Model tested**	**Loglikelihood**	**BIC**	**SS- BIC**	**AIC**	**Entropy**
1 Class	-3546.18	7230.20	7150.95	7142.36	
2 Class	-2785.57	5852.33	5690.66	5673.15	0.92
3 Class	-2584.99	5594.51	5350.42	5323.98	0.94
4 Class	-2500.50	5568.87	5242.36	5206.99	0.94
5 Class	-2438.93	5589.10	5180.16	5135.86	0.92
6 class	-2392.80	5640.18	5148.83	5095.60	0.94

**Table 4:** Probability of item endorsement for PTSD and Grief symptoms for 4-class solution

	**Overall symptom frequency**		**PTSD/PGD (Class 1) 16%**		**PTSD (Class 2) 25%**		**PGD (Class 3) 17%**		**Resilient (Class 4) 43%**	
	**N**	**%**	**Probability**	**SE**	**Probability**	**SE**	**Probability**	**SE**	**Probability**	**SE**
PTSD Symptoms – re-experiencing										
Intrusive memories	125	50.40%	**1.00**	**0.00**	**0.75**	**0.07**	**0.65**	**0.09**	0.14	0.05
Nightmares	85	34.27%	**0.77**	**0.07**	0.56	0.08	0.15	0.07	0.01	0.02
Flashbacks	72	29.03%	**0.85**	**0.06**	0.56	0.09	0.33	0.33	0.04	0.02
Psychological or physiological distress to reminders	46	18.55%	**0.87**	**0.06**	**0.69**	**0.08**	0.56	0.56	0.18	0.04
PTSD symptoms - avoidance										
Avoid thoughts	32	12.90%	**0.90**	**0.05**	**0.63**	**0.08**	0.58	0.10	0.12	0.03
Avoid activities	103	41.53%	**0.78**	**0.07**	**0.77**	**0.06**	0.49	0.11	0.21	0.05
PTSD symptoms - numbing										
Amnesia	88	35.48%	0.50	0.10	0.37	0.07	0.11	0.05	0.03	0.02
Loss of interest	99	39.92%	**0.73**	**0.09**	0.43	0.07	0.07	0.05	0.03	
Feeling detached	73	29.44%	**0.73**	**0.08**	0.27	0.08	0.03	0.03	0.01	0.01
Restricted affect	122	49.19%	0.53	0.09	0.13	0.05	0.10	0.05	0.00	0.00
Foreshortened future	117	47.12%	**0.69**	**0.08**	0.33	0.08	0.10	0.06	0.03	0.02
PTSD Symptoms – hyperarousal										
Insomnia	48	19.35%	**0.90**	**0.05**	**0.66**	**0.08**	0.42	0.09	0.06	0.03
Irritability	58	23.39%	**0.90**	**0.05**	**0.89**	**0.05**	0.45	1.00	0.15	0.05
Difficulty concentrating	54	21.77%	**0.81**	**0.07**	**0.74**	**0.06**	0.17	0.08	0.05	0.03
Hypervigilance	110	44.35%	**0.79**	**0.07**	0.55	0.09	0.19	0.08	0.01	0.01
Startle response	116	46.77%	**0.92**	**0.05**	**0.71**	**0.08**	0.44	0.09	0.09	0.04
PGD symptoms										
Longing or yearning	141	56.85%	**0.94**	**0.05**	0.49	0.07	**1.00**	**0.00**	0.32	0.05
Difficulty accepting death	92	37.10%	**0.90**	**0.05**	0.33	0.10	**0.75**	**0.09**	0.08	0.05
Difficulty trusting others	43	17.34%	**0.60**	**0.10**	0.13	0.06	0.28	0.09	0.02	0.01
Bitterness	114	45.97%	**1.00**	**0.00**	0.37	0.10	**1.00**	**0.00**	0.14	0.04
Difficulty moving on	40	16.13%	**0.79**	**0.10**	0.02	0.02	0.20	0.07	0.00	0.00
Numbing	29	11.69%	**0.71**	**0.10**	0.02	0.02	0.00	0.00	0.00	0.00
Emptiness	50	20.16%	**0.84**	**0.09**	0.06	0.03	0.38	0.09	0.01	0.01
No meaning	31	12.50%	0.58	0.10	0.02	0.02	0.42	0.07	0.00	0.00
Jumpy	71	28.63%	**0.95**	**0.04**	0.29	0.10	0.42	1.00	0.01	0.01

*Values in bold indicate that individuals in this class had a high probability of reporting this symptom (≥0.60).

Class 1 (combined PTSD/PGD, 16%) evidenced high probabilities of almost all PTSD and PGD symptoms, and moderate probabilities of the PTSD symptoms of psychogenic amnesia and restricted affect, and the PGD symptom loss of meaning.

Class 2 (predominantly PTSD, 25%) evidenced high probabilities of the following PTSD symptoms: intrusive memories, distress to reminders, avoidance of thoughts and activities, and all hyperarousal symptoms (except hypervigilance). This class evidenced moderate probabilities for all remaining PTSD symptoms except restricted affect, which had a low probability. This class evidenced moderate probabilities of the following PGD symptoms: longing/yearning, difficulty accepting the death, bitterness, and feeling jumpy; and low probability of all other PGD symptoms.

Class 3 (predominantly PGD, 17%) evidenced high probabilities for the following PGD symptoms: longing/yearning, difficulty accepting the death, and bitterness. All other PGD symptoms were moderately probable, except for numbing. This class also evidenced a high probability of the PTSD symptom of intrusive memories, and moderate probabilities of: nightmares, flashbacks, distress to reminders, avoidance of thoughts and feelings, and all hyperarousal symptoms. This class evidenced low probability of all numbing symptoms.

Class 4 (Resilient, 43%) evidenced low probabilities for the majority of PTSD and PGD symptoms with the exception of PTSD symptoms of distress to reminders, avoidance of activities, and irritability; and PGD symptoms of as well as longing/yearning, which evidenced moderate probabilities.

### Predictors of class membership

A multinomial logistic regression was conducted to examine predictors of class membership (see Table 5). Compared to those in the resilient class, individuals in the combined PTSD/PGD class were more likely to be female, to have been exposed to more types of detention/abuse traumas and traumatic losses, and to report greater difficulties associated with loss of culture and support. Compared to those in the resilient class, individuals in the PTSD class were more likely to be older, to have experienced more types of detention/abuse trauma, and to report more difficulties associated with loss of culture and support. Compared to those in the resilient class, those in the PGD class were more likely to be older, to have reported exposure to more types of detention/abuse trauma and to have experienced greater adaptation difficulties.

**Table 5:** Multinomial logistic regression predicting class membership

	**B**	**SE**	**Exp (B)**	**95% Confidence interval**	***p***
Class 1 PTSD/PGD (vs. Class 4 Resilient)					
Age	0.04	0.02	1.04	0.99 – 1.08	0.09
Gender	1.35	0.66	3.87	1.07- 13.95	0.04
English proficiency	-0.282	0.18	0.75	0.54- 1.06	0.11
Detention and abuse	0.70	0.23	2.01	1.27- 3.16	<0.001
Traumatic loss	0.63	0.28	1.88	1.09- 3.25	0.02
Exposure to conflict	-0.01	0.28	0.99	0.57- 1.71	0.98
Adaptation difficulties	0.17	0.32	1.18	0.63- 2.20	0.60
Threat to family	0.60	0.41	1.82	0.82- 4.07	0.14
Residency determination difficulties	0.56	0.69	1.75	0.45- 6.76	0.42
Healthcare, welfare and asylum difficulties	0.13	0.34	1.14	0.59- 2.21	0.70
Loss of culture and support	0.71	0.19	2.03	1.39- 2.95	<0.001
Class 2 PTSD (vs. Class 4 Resilient)					
Age	0.04	0.018	1.04	1.00- 1.08	0.04
Gender	0.57	0.52	1.77	0.63- 4.94	0.28
English proficiency	-0.10	0.14	0.91	0.68 – 1.20	0.50
Detention and abuse	0.50	0.20	1.65	1.11- 2.46	0.01
Traumatic loss	0.28	0.22	1.32	0.85- 2.05	0.22
Exposure to conflict	0.04	0.22	1.04	0.67- 1.61	0.86
Adaptation difficulties	0.43	0.25	1.54	0.95- 2.49	0.08
Threat to family	0.27	0.32	1.31	0.70- 2.45	0.40
Residency determination difficulties	0.55	0.62	1.74	0.52- 5.85	0.37
Healthcare, welfare and asylum difficulties	0.11	0.28	1.11	0.65- 1.90	0.71
Loss of culture and support	0.40	0.43	1.50	0.64- 3.48	0.01
Class 3 PGD (vs. Class 4 Resilient)					
Age	0.04	0.02	1.04	1.00- 1.08	0.03
Gender	0.67	0.56	1.96	0.65- 5.87	0.23
English proficiency	-0.12	0.15	0.89	0.66- 1.19	0.42
Detention and abuse	0.53	0.21	1.69	1.11- 2.58	0.01
Traumatic loss	0.24	0.24	1.27	0.79- 2.04	0.33
Exposure to conflict	-0.31	0.25	0.73	0.44- 1.20	0.22
Adaptation difficulties	0.68	0.25	1.97	1.20- 3.24	0.01
Threat to family	0.42	0.34	1.53	0.78- 2.97	0.21
Residency determination difficulties	0.48	0.64	1.62	0.46- 5.67	0.45
Healthcare, welfare and asylum difficulties	-0.03	0.29	0.97	0.55- 1.73	0.92
Loss of culture and support	0.24	0.24	1.27	0.79- 2.04	0.33

### Association between class membership and depression symptoms and diagnosis

A multinomial logistic regression indicated that individuals in all symptom classes evidenced significantly higher levels of depression symptoms compared to the resilient class; combined PTSD/PGD Class: B = 5.83, SE = 0.66, *p* < .001, OR = 340.74, 95% Confidence Interval [CI] = 93.73-1238.76); predominantly PTSD Class: B = 3.86, SE = .50, *p* < .001, OR = 47.23, 95% CI = 17.78-125.49); Predominantly PGD Class: B = 2.70, SE = 0.46, *p* < .001, OR = 14.92, 95% CI = 6.10-37.04. Frequencies of depression diagnosis according to class membership were as follows: combined PTSD/PGD Class: N = 37 (97.4%); predominantly PTSD class: N = 49 (81.7%); predominantly PGD class: N = 30 (75%), Resilient class: N = 19 (17.3%).

## Discussion

This study employed LCA to investigate symptom profiles of PGD and PTSD amongst resettled refugees dually exposed to trauma and loss. Four distinct symptom profiles emerged from the data: a combined PTSD/PGD class, a predominantly PTSD class, a predominantly PGD class, and a Resilient class. These findings are novel as they demonstrate that there are divergent PTSD and PGD symptom profiles in a highly traumatized conflict-affected sample also exposed to significant loss. Further, we found evidence that there are differential pathways to these PTSD and PGD subgroups related to the specific nature of the trauma and post-displacement refugee experience.

The emergence of a combined PTSD/PGD class in this sample extends on previous latent class investigations that have documented pervasive disturbance classes in civilian and military populations across PTSD symptoms [13,25]. This suggests that there is a group of refugees who exhibit pervasive distress across symptom types, rather than being characterized by a particular diagnostic category of symptoms such as PTSD or PGD. This is further supported by the finding that participants in this class were much more likely than those in the resilient class to experience elevated symptoms of depression.

The identification of the predominantly PTSD and predominantly PGD classes of participants is novel as it indicates that there are distinctive symptom profiles in distressed sub-groups of a population who have survived mass trauma combined with significant loss. Those in the predominantly PGD class evidenced high probabilities of key PGD symptoms including longing/yearning for the deceased, difficulty accepting the death, and bitterness. A key question is the extent to which the diagnostic criteria for PGD proposed for ICD-11 are universally applicable across cultural groups. While certain key PGD symptoms were highly prevalent in the PGD class in this study, other symptoms evidenced low probabilities, suggesting that not all PGD symptoms are equally pertinent to trauma- and loss-exposed individuals.

Culture and context play a strong role in shaping grief reactions following loss [26]. The finding that bitterness (which can be defined as feeling angry, insulted, or let down, and is associated with feelings of revenge and helplessness [6,9,27]) was highly endorsed in the PGD class in this study is consistent with other research linking PGD and bitterness following exposure to war [9]. This symptom may be an important marker for distress when loss is experienced in the context of violence. Difficulty accepting the loss may also be particularly salient following sudden and violent losses in conflict-affected settings, given it is often difficult to perform important cultural or religious rituals that represent transitional steps to accepting the loss [28]. These findings highlight the key importance of considering cultural and contextual factors when assessing prolonged grief reactions.

It is notable that the resilient class comprised nearly half of the sample. Accordingly, there is considerable research indicating that the majority of people exposed to trauma and loss recover naturally over time and do not report significant ongoing psychopathology [29,30]. This has important public health implications as it suggests that adaptation following exposure to trauma and loss is the normative response, even in the context of persecution and mass violence.

The finding that types of refugee experiences differentially predict class membership suggests that there are differing pathways to psychopathology, even within populations who are universally exposed to loss and trauma. Traumatic events characterized by detention and abuse were associated with membership in all symptomatic classes, which is consistent with much evidence of the dosage relationship between quantity of trauma exposure and risk of mental disorder [31,32]. In contrast, high levels of exposure to traumatic loss were associated only with membership in the combined PTSD/PGD class relative to the resilient class. This indicates that events characterized by both trauma and loss contribute uniquely to a psychological profile that encompasses both PTSD and PGD symptoms. This is consistent with Neria and Litz’s assertion that concurrent exposure to trauma and loss creates a “dual emotional burden” [33]. The combination of traumatic and loss-related aspects of this experience may yield both fear-related symptoms (i.e., PTSD symptoms) and grief reactions. It is notable that traumatic loss did not predict membership in the predominantly PGD class. As all individuals in the current sample had been exposed to the loss of a loved one, it may that it was the traumatic nature of these losses (e.g., witnessing the murder death of a loved one) that specifically contributed to membership in the combined PTSD/PGD classes. Thus, experiencing a traumatic event and a loss concurrently appears to have an especially deleterious impact on the mental health of the trauma survivor, leading to high probability of membership in the PTSD/PGD class.

Loss of culture and support was associated with membership in the PTSD/PGD and PTSD classes. Many Mandaeans who participated in this study expressed concern over the loss of culture and traditions, with difficulties getting access to appropriate foods and/or places where they could conduct religious ceremonies, as well as being refused permission to perform necessary religious rituals, being examples of salient stressors. This is consistent with research indicating that fear of cultural extinction was associated with higher levels of PTSD reactions in Mandaean refugees [34], who face the real possibility that their culture will cease to exist within generations as a result of systematic persecution and religious dictates forbidding marriage with non-Mandaeans [35]. Thus, difficulties in maintaining cultural traditions may be associated with greater perceived threat to culture, therefore contributing to PTSD symptomatology. This is also consistent with the construct of cultural bereavement which integrates the broader impact of refugee experiences such as trauma exposure and loss of culture with mental health symptoms [36]. Adaptation-related living difficulties were specifically associated with membership in the PGD class. These can be conceptualized as forms of loss in relation to lifestyle, status, and support networks. PGD is compounded by perceived lack of social support [37], and it is probable that separation from traditional supports in the relocation process contributes to persistent grief reactions. It is somewhat surprising that loss of culture and support did not predict membership in the PGD only class, considering the strong documented relationship between PGD and lack of social support [38,39]. These findings suggest that there is a unique relationship between loss of culture and support and PTSD reactions in this sample. Further research should elucidate this relationship.

It is notable that English proficiency did not predict membership in any of the symptomatic classes, despite research suggesting that proficiency in the language of the host country is associated with poorer mental health outcomes [40,41]. It may be that the difficulties encompassed in the adaptation subscale (i.e., communication difficulties) encapsulated the effect of poor English proficiency. It is also interesting to note that older age significantly predicted membership in the PTSD only and PGD only classes, and marginally significantly predicted membership in the combined PTSD/PGD classes. This is in contrast to prior research which has suggested that younger refugees tend to report higher levels of psychological symptoms [17,42]. One possibility is that, in this sample, older refugees had been exposed to more traumatic events, and accordingly were more likely to be in the symptomatic classes. Finally, it was somewhat surprising to note that difficulties related to the asylum process did not predict group membership in this study considering the body of research indicating that asylum-related factors are strongly related to mental health outcomes [14,43,44]. One possible explanation for this is that, at the time of this study, all participants had obtained permanent visa status in Australia. This, it may be that the acute impact of mental health problems related to insecure visa status were not observed in the results.

The present study has several limitations. As no census information is available on the Mandaean community, we had to rely on community lists provided by Mandaean leaders for recruitment for this study. It may be that individuals with more or less severe psychological distress were more likely to be in contact with leaders, and were thus overrepresented in this sample. We did not assess the number of losses the individual had experienced, so were unable to investigate whether there is a dose–response effect between loss and PGD/PTSD reactions. We did not assess anxiety reactions outside of the PTSD criteria so were unable to examine the extent to which these were related to class membership. Further, while it would be informative to consider the symptom profiles that emerge when including depression reactions in the LCA model, our sample size precluded this extended analysis. Instruments used in this study were not specifically developed for or validated with Mandaean refugees; for example, the Inventory of Complicated Grief was originally developed in North America [45], and the HTQ and HSCL were developed/adapted for use with refugees from South East Asia [16,19]. However, these measures have strong psychometric properties and have been used across multiple cultures, including with groups from the Middle East [46-48]. Further, there are several other contextual factors that may impact on symptom profiles that were not examined in this study, for example, culture, religion, relationship to the deceased, whether the individual was internally displaced, displaced to a refugee camp, or held in immigration detention. Further research should investigate the effect of these and other factors on symptom profiles.

Findings from the present study have important clinical implications for those working with survivors of trauma and loss. The finding that there are distinctive symptom profiles that are associated with specific experiences underscores the need to differentially assess and manage psychological distress in patients exposed to both trauma and loss. For example, it may be possible to develop a screening instrument that indexes key reactions and experiences that are likely to lead to specific types of psychopathology. Further, the development of interventions that target differential symptom profiles should be prioritized. While there is a growing body of evidence supporting the use of trauma-focused interventions for the treatment of posttraumatic stress responses in refugees [49], there is an urgent need for further research investigating the treatment of prolonged grief symptoms (both in alone and in combination with PTSD symptoms) in refugees dually exposed to trauma and loss. Findings from the current study suggest that, in many survivors of trauma and loss, psychological distress is not pervasive, but manifests in particular symptom groups. As such, interventions tailored to the patient’s specific needs may be more effective than universal treatments that indiscriminately target psychological distress in these groups. Findings from this study may also inform the understanding of and development of interventions for other groups dually affected by multiple traumas and losses, for example, military populations, war-affected civilians, internally displaced persons, and individuals exposed to traumatic loss. Further research should be conducted to determine whether these symptom profiles extend to these other populations, and the specific characteristics and experiences that predict specific symptom clusters in these groups.

## Conclusions

Conflict-affected populations are at heightened risk for the development of disabling mental disorders like PTSD and PGD as a result of exposure to trauma and loss. The current findings show that distinct PTSD or PGD symptom profiles emerge even in individuals who have experienced loss in the context of mass trauma and persecution. These symptom profiles are associated with exposure to different types of refugee experiences, with exposure to traumatic loss predicting membership in a group characterized by high levels of both PTSD and PGD symptoms, while loss of culture and support predicts membership in a group with high levels of PTSD symptoms, and adaptation difficulties predict group membership associated with high levels of PGD symptoms. These results underscore the importance of identifying specific symptom profiles in individuals exposed to both trauma and loss. This may facilitate the development of intervention strategies that target specific types of distress in survivors of persecution and conflict.

## Competing interests

The authors declare that they have no competing interests.

## Authors’ contributions

AN was involved in the conception and design of the study, the acquisition, analysis and interpretation of the data, and the drafting of the manuscript. She is accountable for all aspects of the work. BL was involved in the analysis and interpretation of the data and the drafting of the manuscript. FM was involved in the analysis and interpretation of the data and the drafting of the manuscript. ZS was involved in the conception and design of the study, the acquisition, analysis and interpretation of the data, and the drafting of the manuscript. DS was involved in the conception and design of the study and the drafting of the manuscript. RB was involved in the conception and design of the study, the acquisition, analysis and interpretation of the data, and the drafting of the manuscript. All authors read and approved the final manuscript.

## Pre-publication history

The pre-publication history for this paper can be accessed here:

http://www.biomedcentral.com/1471-244X/14/106/prepub
